# Genome-enabled studies of anaerobic, nitrate-dependent iron oxidation in the chemolithoautotrophic bacterium *Thiobacillus denitrificans*

**DOI:** 10.3389/fmicb.2013.00249

**Published:** 2013-08-27

**Authors:** Harry R. Beller, Peng Zhou, Tina C. Legler, Anu Chakicherla, Staci Kane, Tracy E. Letain, Peggy A. O’Day

**Affiliations:** ^1^Earth Sciences Division, Lawrence Berkeley National LaboratoryBerkeley, CA, USA; ^2^University of CaliforniaMerced, CA, USA; ^3^Lawrence Livermore National LaboratoryLivermore, CA, USA

**Keywords:** iron oxidation, nitrate-dependent, anaerobic, *Thiobacillus denitrificans*, chemolithoautotrophic, reverse electron transfer

## Abstract

*Thiobacillus denitrificans* is a chemolithoautotrophic bacterium capable of anaerobic, nitrate-dependent U(IV) and Fe(II) oxidation, both of which can strongly influence the long-term efficacy of *in situ* reductive immobilization of uranium in contaminated aquifers. We previously identified two *c*-type cytochromes involved in nitrate-dependent U(IV) oxidation in *T. denitrificans* and hypothesized that *c*-type cytochromes would also catalyze Fe(II) oxidation, as they have been found to play this role in anaerobic phototrophic Fe(II)-oxidizing bacteria. Here we report on efforts to identify genes associated with nitrate-dependent Fe(II) oxidation, namely (a) whole-genome transcriptional studies [using FeCO_3_, Fe^2^^+^, and U(IV) oxides as electron donors under denitrifying conditions], (b) Fe(II) oxidation assays performed with knockout mutants targeting primarily highly expressed or upregulated *c*-type cytochromes, and (c) random transposon-mutagenesis studies with screening for Fe(II) oxidation. Assays of mutants for 26 target genes, most of which were *c*-type cytochromes, indicated that none of the mutants tested were significantly defective in nitrate-dependent Fe(II) oxidation. The non-defective mutants included the *c*_1_-cytochrome subunit of the cytochrome *bc*_1_ complex (complex III), which has relevance to a previously proposed role for this complex in nitrate-dependent Fe(II) oxidation and to current concepts of reverse electron transfer. A transposon mutant with a disrupted gene associated with NADH:ubiquinone oxidoreductase (complex I) was ~35% defective relative to the wild-type strain; this strain was similarly defective in nitrate reduction with thiosulfate as the electron donor. Overall, our results indicate that nitrate-dependent Fe(II) oxidation in *T. denitrificans* is not catalyzed by the same *c*-type cytochromes involved in U(IV) oxidation, nor have other *c*-type cytochromes yet been implicated in the process.

## INTRODUCTION

*In situ* microbial reductive immobilization of radionuclides in aquifers is a remedial approach that has been the subject of considerable interest since the 1990s. The essence of this approach is that many radionuclides of concern are redox-active and are less soluble in their reduced form, and thus can be immobilized in aquifers *via* microbially mediated reduction under anaerobic conditions. For example, U(VI) in the form of $U^{VI}O_{2}^{2+}$ (aq) and its complexes is relatively water soluble, whereas the mineral uraninite [UO_2_(s) or other amorphous or nanocrystalline U(IV)-oxide phases], typically formed by U(VI)-reducing bacteria (Lovley and Phillips, 1992b; Fredrickson et al., 2000), has very low solubility. A range of bacteria has been shown to be capable of direct microbial reduction of uranium, including *Geobacter metallireducens*, *Shewanella oneidensis*, *Desulfovibrio desulfuricans*, *D. vulgaris*, and many others (Lovley et al., 1991, 1993; Lovley and Phillips, 1992a, b). However, recent studies have suggested that microbially mediated, nitrate-dependent U(IV) oxidation under anaerobic conditions could complicate efforts at long-term reductive immobilization. Nitrate-dependent oxidation is of particular relevance because nitrate is a common co-contaminant with uranium at U.S. Department of Energy (DOE) sites (Riley and Zachara, 1992). Finneran et al. (2002) showed that nitrate-grown, but not Fe(III)-grown, cells of *G. metallireducens* carried out nitrate-dependent U(IV) oxidation in anaerobic cell suspensions amended with UBr_4_, a soluble form of U(IV). Beller (2005) showed that the widely distributed, chemolithoautotrophic bacterium *T. denitrificans* is capable of anaerobic, nitrate-dependent oxidative dissolution of synthetic and biogenic U(IV) oxides, such as uraninite. In addition to controlled cell suspension studies with bacteria such as *G. metallireducens* and *T. denitrificans*, there is field evidence indicating the real-world relevance of nitrate-dependent oxidative mobilization of uranium. Senko et al. (2002) observed nitrate-dependent uranium solubilization during a push-pull (i.e., single-well) field study. In this study, much of the uranium that was previously immobilized in the aquifer was re-mobilized over a period of 1–2 weeks after addition of nitrate to the groundwater; the bacterial species and biogeochemical mechanisms involved were not elucidated in that study.

Furthermore, nitrate-dependent Fe(II) oxidation appears to enhance U(IV) oxidation, as has been observed for *G. metallireducens* (Finneran et al., 2002), an isolate from DOE’s FRC site (Senko et al., 2005a), and *T. denitrificans* (H. R. Beller, unpublished data). The reason for the enhancement of U(IV) oxidation in the presence of nitrate-dependent Fe(II) oxidation has not been definitively shown. However, there is a growing body of evidence that substantiates the ability of Fe(III)-(hydr)oxide solids in the presence of dissolved Fe^2^^+^ and nitrite to oxidize UO_2_(s) to aqueous uranyl ($U^{VI}O_{2}^{2+}$; Finneran et al., 2002; Sani et al., 2005; Senko et al., 2005a, b, 2007; Wan et al., 2005; Ginder-Vogel et al., 2006; Tokunaga et al., 2008). For example, Senko et al. (2005a) reported UO_2_(s) oxidation in the presence of Fe(III) minerals, including ferrihydrite [Fe(OH)_3_(s)] and a poorly crystalline Fe(III) solid resulting from the abiotic oxidation of Fe(II) by nitrite.

Microbial Fe(II) oxidation, including anaerobic, nitrate-dependent Fe(II) oxidation, has been reviewed by a number of authors in recent years (Weber et al., 2006a; Bird et al., 2011; Hedrich et al., 2011; Roden, 2012; Ilbert and Bonnefoy, 2013) and will not be covered in detail here. The initial report of nitrate-dependent Fe(II) oxidation by Straub et al. (1996) included the observation that *T. denitrificans* could couple denitrification to anaerobic oxidation of ferrous iron in common minerals, such as FeS. Known representatives of the anaerobic, nitrate-dependent Fe(II)-oxidizing bacteria fall within several classes of the proteobacteria, in particular the β-proteobacteria (e.g., *T. denitrificans*, *Pseudogulbenkiania* sp. 2002, *Azospira oryzae* strain PS, *Aquabacterium* sp BrG2, *Acidovorax* sp BrG1, and *Acidovorax ebreus* TPSY), but also the α-proteobacteria (*Paracoccus ferrooxidans* strain BDN-1), γ-proteobacteria (e.g., *Thermomonas* sp. BrG3), and the δ-proteobacteria (*G. metallireducens*; Hedrich et al., 2011). Very few of the studied anaerobic, nitrate-dependent Fe(II)-oxidizing microbial isolates are able to carry out this activity under strictly autotrophic growth conditions; these include *Pseudogulbenkiania* sp. 2002 (Weber et al., 2006b, 2009) and the hyperthermophilic archaeum *Ferroglobus placidus* (Hafenbradl et al., 1996). Although *T. denitrificans*, an obligate chemolithoautotrophic bacterium, was reported to carry out nitrate-dependent Fe(II) oxidation (Straub et al., 1996), energy conservation and growth were not demonstrated. As has been noted previously (Weber et al., 2006a; Picardal, 2012), many of the nitrate-dependent, Fe(II)-oxidizing cultures described to date are organotrophic (or at least mixotrophic) rather than autotrophic, and the use of organic compounds (such as acetate) as carbon sources and electron donors complicates the interpretation of their physiology and metabolism. In this sense, the lithoautotroph *T. denitrificans* represents a more simple experimental system for the study of nitrate-dependent Fe(II) oxidation.

Among anaerobic, Fe(II)-oxidizing bacteria, the primary enzymes associated with catalysis of Fe(II) oxidation have only been identified in anoxygenic, phototrophic bacteria, not in any nitrate-reducing, Fe(II)-oxidizing strains to date (Bird et al., 2011; Ilbert and Bonnefoy, 2013). In the phototroph *Rhodopseudomonas palustris *TIE-1, the *pioABC* operon has been demonstrated to be essential to Fe(II) oxidation (Jiao and Newman, 2007). The proteins encoded by *pioABC* include PioA, a periplasmic, decaheme, *c*-type cytochrome, PioB, an outer-membrane (OM) porin, and PioC, a periplasmic high-potential iron-sulfur protein (HiPIP; Jiao and Newman, 2007). Notably, PioA and PioB share homology with MtrA and MtrB, respectively, in *Shewanella oneidensis* MR-1, which have been associated with Fe(III) reduction in that bacterium. In the phototroph *Rhodobacter *strain SW2, the *foxEYZ* operon has been linked to Fe(II) oxidation by heterologous expression in the related and genetically tractable *Rhodobacter capsulatus* SB1003 (Croal et al., 2007). The proteins encoded by *foxEYZ* include FoxE, a diheme *c*-type cytochrome (Saraiva et al., 2012), FoxY, a predicted redox cofactor pyrroloquinoline quinone, and FoxZ, a predicted inner-membrane transport protein. Although the PioABC and FoxEYZ systems are not homologous, both contain *c*-type cytochromes as essential components. Considering the thermodynamic constraints on compounds that could serve as physiological electron acceptors for Fe(II) oxidation, the involvement of *c*-type cytochromes is not surprising. To illustrate, the reduction potential for the Fe(OH)_3_/FeCO_3_ couple (~0.2 V; Hedrich et al., 2011) is high relative to common electron carriers in the cell, such as ferredoxin, NAD^+^, FAD, menaquinone, and ubiquinone, which collectively fall in the reduction potential range of -0.4 to +0.11 V (Thauer et al., 1977). In fact, *c*-type and *a*-type cytochromes are among the few common electron carriers with sufficiently high reduction potentials to serve as electron acceptors for Fe(II). Another reason for *c*-type cytochromes to be candidates for nitrate-dependent Fe(II) oxidation in *T. denitrificans* is that two diheme, *c*-type cytochromes were previously shown to be associated with nitrate-dependent U(IV) oxidation in that species (Beller et al., 2009).

In this article, we explore anaerobic, nitrate-dependent Fe(II) oxidation in the widespread, obligately chemolithoautotrophic bacterium *T. denitrificans*, which has also been shown to be capable of anaerobic, nitrate-dependent U(IV) oxidation. Almost nothing is known about the underlying biochemistry and genetics of nitrate-dependent Fe(II) and U(IV) oxidation in any bacteria or archaea. Inasmuch as *T. denitrificans* already has a sequenced genome (Beller et al., 2006a), genetic system (Letain et al., 2007; Beller et al., 2012), and custom-designed gene expression microarrays (Beller et al., 2006b), it served as a good subject for genome-enabled studies. We report on whole-genome transcriptional studies of *T. denitrificans* comparing gene expression under nitrate-dependent Fe(II)-, U(IV)-, and thiosulfate (control)-oxidizing conditions, targeted gene knockouts based on these results (reverse genetics), as well as random transposon mutagenesis studies (forward genetics). To our knowledge, this is the most extensive investigation to date of enzymes involved in anaerobic, nitrate-dependent Fe(II) oxidation.

## MATERIALS AND METHODS

### ANALYTICAL METHODS

Thiosulfate, sulfate, nitrate, and nitrite concentrations were measured by ion chromatography (IC) using a Model DX 500 IC (Dionex Corporation, Sunnyvale, CA, USA) or a Model ICS-2000 IC (Dionex) with micromembrane suppression and electrochemical conductivity detection (Beller, 2005; Han et al., 2010). Quantification relied on external standards using a 3-point calibration.

The microplate assay used for Fe(II) analysis was described previously by Beller et al. (2013). Microplates (96-well) that had been stored in an anaerobic glove box for at least one day were amended with 90 μL of 1N HCl, and a 10 μL cell suspension sample was added to the HCl immediately after sampling. Then, 100 μL of Ferrozine solution (1 g/L Ferrozine, 500 g/L ammonium acetate) was added to the acidified sample. After a 10-min incubation, absorbance at 570 nm was measured using a Model 550 microplate reader (Bio-Rad, Hercules, CA, USA). Fe(II) standards (0.2, 0.5, 1, and 2 mM ferrous ammonium sulfate hexahydrate in 1 N HCl) were included on each microtiter plate.

Highly purified water (18 Ω resistance) obtained from a Milli-Q Biocel system (Millipore, Bedford, MA, USA) was used to prepare all aqueous solutions described in this article.

### WHOLE-CELL SUSPENSION ASSAYS FOR NITRATE-DEPENDENT Fe(II) OXIDATION

*In vivo* assays of nitrate-dependent Fe(II) oxidation by wild-type *T. denitrificans* (ATCC strain 25259, obtained from the American type culture collection) and various mutant strains were conducted under strictly anaerobic conditions in an anaerobic glove box. Cells (typically 400 mL) were cultivated at 30°C as described previously (Beller, 2005) with growth medium that contained 20 mM thiosulfate, 20 mM nitrate, 30 mM bicarbonate (pH ~7), and kanamycin and/or gentamicin (as appropriate). Cells were harvested anaerobically by centrifugation (13,400 × *g*, 20°C, 15 min), washed by resuspension in 200 mL of basal anaerobic buffer described previously [Beller, 2005; NH_4_Cl (18.7 mM), KH_2_PO_4_ (1.5 mM), NaHCO_3_ (30 mM), MgSO_4_ (3.25 mM)], centrifuged again (13,400 × *g*, 20°C, 10 min), and resuspended in a volume of basal anaerobic buffer (typically, ~2 mL) to result in an OD_600_ ≈ 8. (An OD_600_ value of 1 corresponds to approximately 9 × 10^8^ cells per mL or 0.19 mg protein per mL.) Cell suspensions were mixed with an equal volume of anaerobic, filtered assay solution, which consisted of basal buffer amended with 3 mM FeSO_4_, 2 mM KNO_3_, and 20 mM trisodium nitrilotriacetate (NTA). The NTA was added to prevent encrustation of Fe(III) precipitates on cells (Jiao and Newman, 2007). The assays (200 μL total volume) were performed in 96-well microplates under static conditions. No-nitrate, no-cell, and no-iron controls were performed in a similar manner, except that KNO_3_, cells, or FeSO_4_ were replaced with anaerobic reagent water. Concentrations of Fe(II), nitrate, and nitrite were measured at 0, 1, 2.5, and 5 h by the Ferrozine microplate and IC methods described above. Fe(II) oxidation assays were performed with biological triplicates as well as analytical triplicates for the microplate assays.

For selected strains, positive controls to assess batch-specific denitrification activity of *T. denitrificans* cells were carried out in the growth medium (i.e., with thiosulfate as the electron donor) and received the same inoculum (in terms of concentration of cells) as cultures assayed for Fe(II) oxidation (Beller, 2005).

Abiotic tests of nitrite redox interactions with Fe(II) were conducted as described above except that KNO_3_ was replaced with NaNO_2_ at a final concentration of 150 μM and no cells were added.

### TESTS FOR GROWTH OF *T. denitrificans* USING Fe(II) AS THE ELECTRON DONOR

Growth of *T. denitrificans* on Fe(II)-nitrate medium was tested anaerobically under a 90% N_2_–10% CO_2_ atmosphere at 30°C. The differences between the medium used for these experiments and standard growth medium (Beller, 2005) were as follows: 10 mM FeSO_4_ replaced 20 mM thiosulfate as the electron donor, and the concentration of KNO_3_ was lowered from 20 to 2.5 mM. The inoculum was 10% (vol/vol) thiosulfate-grown cells that were washed with growth medium lacking thiosulfate and nitrate. Three biological replicates were tested along with controls lacking either FeSO_4_ or KNO_3_. Consumption of Fe(II) was measured with the Ferrozine assay described above and protein concentration was determined with a Quick Start Bradford Protein Assay Kit (Bio-Rad).

### EXPOSURE CONDITIONS FOR GENE EXPRESSION MICROARRAYS

To represent gene expression under denitrifying conditions with various electron donors, wild-type *T. denitrificans* was cultivated at 30°C under strictly anaerobic conditions as described previously (Beller, 2005) with growth medium that contained 20 mM thiosulfate, 20 mM nitrate, and 30 mM bicarbonate (pH ~7). For exposure immediately before harvesting of RNA, 1,200 mL of cells in late exponential phase (1 × 10^8^ to 2 × 10^8^ cells/mL) were harvested anaerobically by centrifugation (13,400 × *g*, 15°C, 10 min), washed by resuspension in 75 mL of basal anaerobic buffer described previously (Beller, 2005), centrifuged again (13,400 × *g*, 15°C, 5 min), resuspended in 4 mL of basal anaerobic buffer, and 1 mL of this cell suspension (containing an average of 4.8 mg protein) was added to 4 or 9 mL of buffer amended with a variety of solutions, described below.

The range of exposure conditions is listed in **Table 1**. With the exception of aerobic, thiosulfate-oxidizing conditions (Group IV; previously described by Beller et al., 2006b), all incubations were under anaerobic, denitrifying conditions in serum vials sealed with butyl rubber stoppers. All materials for manipulating and containing samples were allowed to degas in the anaerobic glove box for at least 2 days. Three or four biological replicates were tested for each condition. For thiosulfate-oxidizing, denitrifying conditions (Groups III, VII, and VIII; **Table 1**), incubations were performed in 10-mL volumes with 20 mM thiosulfate and 20 mM nitrate for 40 min (Groups VII and VIII) or 60 min (Group III). (The 75-mL cell-washing step described above was not used for thiosulfate-oxidizing cells, since they were harvested after growth under the same conditions). For anaerobic incubations conducted in the absence of H_2_ (Groups I and VIII; **Table 1**), sealed serum bottles were vacuum-gassed three times with an anaerobic mixture of 90% N_2_–10% CO_2_, whereas incubations with H_2_ (Groups II, III, V, VI, and VII; **Table 1**) had a mixture of approximately 85% N_2_ – 10% CO_2_ – 5% H_2_ in the headspace. Incubation conditions listed with FeCO_3_ (Groups I and V) contained 3 mM nitrate and 3 mmol/L Fe(II) precipitate; the precipitate was formed 1–2 h before incubation by adding FeSO_4_ to the basal resuspension medium (which contained 30 mM ${HCO}_{3}^{-}$ ) without cells. Equilibrium modeling of the initial assay mixture using TOUGHREACT (Xu et al., 2011) with a modified MinteqA2 v.4 database (U.S. Environmental Protection Agency, 1999) using a revised solubility constant for siderite (FeCO_3_; Preis and Gamsjager, 2002) indicated that FeCO_3_ would constitute >99% of the initial Fe-containing precipitates. Incubation conditions with mostly dissolved Fe(II) (Fe^2^^+^; Group II) contained 3 mM nitrate and 3 mM Fe(II), but were conducted in a 20 mM-MOPS [3-(*N*-morpholino)propanesulfonic acid] buffered medium rather than 30 mM ${HCO}_{3}^{-}$ -buffered medium to preclude precipitation with carbonate. Finally, incubation with U(IV) oxides (Group VI) included 3 mM nitrate and either ~0.225 mmol/L biogenic uraninite (described previously; Beller, 2005; three biological replicates) or ~2 mmol/L synthetic U(IV) oxide slurry (described previously; Beller, 2005; one biological replicate]. All incubations with Fe(II) or U(IV) were carried out in a 5-mL liquid volume for ~200 min. Chemical controls (lacking *T. denitrificans* cells) were conducted for Fe(II) and U(IV) experiments to confirm that oxidation was microbially mediated.

**Table 1:** Exposure conditions forT. denitrificans cells used for gene expression microrrays^a^.

Group	Electron donor	Electron acceptor	pH buffer^b^	GEO^c^ accession number
IV	Thiosulfate, no H_2_	O_2_	Bicarbonate	GSM119288, GSM120794, GSM120797
VIII	Thiosulfate, no H_2_	Nitrate	Bicarbonate	GSM120793, GSM120795, GSM120796
III	Thiosulfate, H_2_	Nitrate	Bicarbonate	GSM1128769-71
VII	Thiosulfate, H_2_	Nitrate	Bicarbonate	GSM1128782-4
II	Fe^2+^ (aq.), H_2_	Nitrate	MOPS	GSM1128766-8
I	FeCO_3_, no H_2_	Nitrate	Bicarbonate	GSM1128763-5
V	FeCO_3_, H_2_	Nitrate	Bicarbonate	GSM1128775-7
VI	UO_2_, H_2_	Nitrate	Bicarbonate	GSM1128778-81

a See text for details. All experiments other than Group IV were conducted with one of three large batches of cells; each batch of cells was split into up to four smaller batches that were exposed to various electron donors (in triplicate).

b Buffers also contained 1.5 mM phosphate, except for Group IV, which contained 70 mM phosphate (Beller etal., 2006b).

c GEO, Gene Expression Omnibus.

The relevant metabolic activity [e.g., as applicable, thiosulfate oxidation to sulfate, nitrate reduction, Fe(II) oxidation to Fe(III), U(IV) oxidation to U(VI)] was confirmed in all anaerobic and aerobic suspensions by sampling each culture twice: immediately upon resuspension and immediately before harvesting for RNA. Previous experiments indicated that the sampling times used for the various conditions were appropriate for capturing ongoing metabolic activity. Analytical methods are described above.

### RNA EXTRACTION FOR MICROARRAYS

Immediately after exposures (i.e., within 15 s), two volumes of RNAprotect (Qiagen, Valencia, CA, USA) were added to each culture. Samples were incubated at room temperature for 12 min, split in half, and centrifuged at 4,000 rpm for 10 min. The supernatant was decanted and the pellet was stored at -20°C until extraction. RNA extraction was carried out with a MasterPure Complete DNA and RNA Purification Kit (Epicentre Biotechnologies, Madison, WI, USA) using a modified protocol. Briefly, the frozen RNA pellet was removed from frozen storage, thawed on ice, and resuspended in 100 μL 5 mM EDTA (ethylenediaminetetraacetic acid) to remove iron from cells exposed to iron. After resuspension of the pellet, 300 μL of lysis solution containing 112 μg proteinase K was added to the cell pellet and the sample was incubated at 65°C for 20–25 min. The sample was placed on ice for 3–5 min and 200 μL of MPC solution was added to precipitate protein. The supernatant was recovered after centrifugation at >10,000 × *g*, at 4°C for 10 min. Nucleic acid was subsequently precipitated from the supernatant after addition of 500 μL 99% isopropanol and centrifugation at >10,000 × *g*, at 4°C for 10 min. The pellet was treated with DNase I for 20 min at 37°C. To this sample was added 200 μL each of 2× T&C lysis solution and MPC solution with vortexing after each addition. The samples were placed on ice for 3–5 min and centrifuged at >10,000 × *g*, at 4°C for 10 min. RNA in the supernatant was recovered by isopropanol precipitation as described. The RNA pellet was washed twice with 75% ethanol, dried briefly, suspended in water, and stored at -80°C until cDNA synthesis. Aliquots were analyzed with a Bioanalyzer (Agilent), which indicated minimal degradation and concentrations ranging from 310 to 2,000 ng/μL. DNA absorbance 260/280 ratios ranged from 1.7 to 2.1.

### PREPARATION OF LABELED cDNA FOR MICROARRAYS AND MICROARRAY HYBRIDIZATION AND SCANNING

cDNA production and labeling, as well as array hybridization and scanning were performed as described previously (Beller et al., 2006b).

### MICROARRAY DATA ANALYSIS

Investigation of reproducible differences between treatments was performed using the Bioconductor R software package. Data were processed using quantile normalization (Bolstad et al., 2003) and background correction was performed using the RMA (Robust Multi-array Average) method. Data were visualized with scatterplots (volcano plots) that plotted the log odds of differential expression versus no differential expression (B-statistic) on the *y*-axis versus the log_2_-fold change between the two experimental conditions (M value) on the *x*-axis. A threshold of significance was set at *P* ≤ 0.0001 (i.e., this was the threshold used for determining differential expression). Volcano plots were generated with the “volcanoplot” function in the Limma package (Linear Models for Microarray Data; Smyth, 2005). Intensities were adjusted to have the same interquartile range. A linear model fit was determined for each gene using the Limma package and lists of genes with the most evidence of differential expression were obtained.

The microarray design for *T. denitrificans* ATCC 25259 has been described elsewhere (Beller et al., 2006b) and is documented in the NCBI GEO database (accession number GPL3977).

### CONSTRUCTION OF MUTANT STRAINS WITH TARGETED MUTATIONS

In some cases (see **Table 2**), targeted mutations were kanamycin (kan)insertion mutations constructed in *T. denitrificans* as described previously (Letain et al., 2007; Beller et al., 2009, 2012). However, for the majority of the targeted mutations, a more rapid, single-step gene replacement approach was performed using a modified version of a technique described elsewhere (Murphy et al., 2000; Lloyd et al., 2003). To illustrate, we describe below how this technique was used to replace the Tbd_0055 gene by homologous recombination. After genomic DNA was extracted using the MasterPure DNA purification kit (Epicentre Biotechnologies), it was used as the template for three primary PCRs performed using *Taq* DNA polymerase (Qiagen; Q-Solution was used for reactions involving *T. denitrificans* genomic DNA):

**Table 2:** Strains, plasmids, and transposons used in this study.

Strain, plasmid, or transposon	Genotype or markers; characteristics and uses	Source or reference
**Strains**		
*Escherichia coli* TOP10	F^-^ *mcrA* Δ*(mrr-hsdRMS-mcrBC)* Φ80*lacZ*Δ*M15* Δ*lacX74* *recA1* araD139 Δ*(ara-leu)7697 galU galK rpsL* (Str^ R^) *endA1 nupG*	Invitrogen
***Thiobacillus denitrificans***		
ATCC 25259	Wild-type	American Type Culture Collection, Manassas, VA, USA
Tbd_0055 mutant	Tbd_0055::kan	This work
Tbd_0070 mutant	Tbd_0070::kan	This work
Tbd_0094 mutant	Tbd_0094::kan	This work
Tbd_0128-Tbd_0129 mutant^a^	Tbd_0128-Tbd_0129::kan	This work
Tbd_0137 mutant^b^	Tbd_0137::kan	This work
Tbd_0137-Tbd_0139 mutant^a^	Tbd_0137-Tbd_0139::kan	This work
Tbd_0146 mutant^b^	Tbd_0146::kan	Beller etal. (2009)
Tbd_0187 mutant^b^	Tbd_0187::kan	Beller etal. (2009)
Tbd_0146, Tbd_0187 mutant^b^	Tbd_0146::kan Tbd_0187::Gen	This work
Tbd_0341 mutant	Tbd_0341::kan	This work
Tbd_0723 mutant^b^	Tbd_0723::kan	Beller etal. (2009)
Tbd_0820 mutant	Tbd_0820::kan	This work
Tbd_0822 mutant	Tbd_0822::kan	This work
Tbd_1357 mutant	Tbd_1357::kan	This work
Tbd_1398 mutant^b^	Tbd_1398::kan	Beller etal. (2009)
Tbd_1741 mutant	Tbd_1741::kan	This work
Tbd_1831 mutant	Tbd_1831::kan	This work
Tbd_1948 mutant^b^	Tbd_1948::kan	Beller etal. (2009)
Tbd_2026-Tbd_2027 mutant^a^	Tbd_2026-Tbd_2027	This work
Tbd_2060 mutant	Tbd_2060::kan	This work
Tbd_2181 mutant	Tbd_2181::kan	This work
Tbd_2545 mutant	Tbd_2545::kan	This work
Tbd_2628 mutant	Tbd_2628::kan	This work
Tbd_2726 mutant^b^	Tbd_2726::kan	Beller etal. (2009)
Tbd_1145 mutant	Tbd_1145::kan	This work
**Plasmids**		
pUC19	pMB1, Amp^R^; cloning vector	
pUC19-Kan-P137A11	pMB1, Amp^R^, Kan^R^; harboring the kanamycin resistance selection marker flanked by genomic fragments from the *nuoD* mutant	This work
pUC19-Tbd0187	pMB1, Amp^R^, pUC19 with Tbd_0187 (-867, +1396) inserted at MCS	Beller etal. (2009)
pUC19-Tbd0187::gent	pUC19-Tbd0187 with Tn-gent inserted in Tbd_0187	This work
pUC19-Tbd0146::kan	pUC19-Tbd0146 with Tn-kan inserted at +33 of Tbd_0146	Beller etal. (2009)
pUC19-Tbd0137	pMB1, Amp^R^, pUC19 with Tbd 0137 inserted at MCS	This work
pUC19-Tbd0137::kan	pUC19-Tbd0137 with Tn-kan inserted in Tbd_0137	This work
**Transposons**		
EZ-Tn5 < KAN-2 > Tnp Transposome Kit	Kan^R^, DNA fragment with kanamycin resistance selection marker located between Mosaic End Tn*5* transposase recognition sequences	Epicentre Biotechnologies
Tn-gent	Gent^R^, DNA fragment with gentamicin resistance selection marker located between Mosaic End Tn5 transposase recognition sequences	Letain etal. (2007)

a Indicates that two or three adjacent genes were replaced, not just a single gene.

b Insertion mutant created as described by Letain etal. (2007). Other mutants listed are gene replacement mutants made as described in Section “Materials and Methods.”

(i) primers Tbd_0055-1 and Tbd_0055-2 (**Table 3**) were used to amplify the upstream sequence of the Tbd_0055 gene using the following conditions: 94°C for 30 s, followed by 35 cycles of 94°C for 30 s, 55°C for 30 s, and 72°C for 1 min and a final extension at 72°C for 10 min. The amplicon was purified with QIAquick PCR Purification Kit (Qiagen) and digested with EcoRI (New England Biolabs, Ipswich, MA, USA), which was introduced with Primer Tbd_0055-2.
(ii) primers Tbd_0055-5 and Tbd_0055-6 (**Table 3**) were used to amplify the downstream sequence of the Tbd_0055 gene (using the same PCR conditions listed above) and the amplicon was purified with QIAquick PCR Purification Kit (Qiagen) and was digested with XbaI (New England Biolabs), which was introduced with Primer Tbd_0055-5;
(iii) primer KO3-EcoRI (which introduced EcoRI) and primer KO4 (**Table 3**) were used to amplify the Kan^ R^ cassette, with the EZ-Tn5 < KAN-2 > Tnp Transposome serving as the template, and the product was purified and digested with EcoRI and XbaI.

**Table 3:** PCR primers used in this study.

Primer	Sequence^a^ (5′–3′)
Tbd_0055-1	ATTCGAGGGCGAAGCCGAAG
Tbd_0055-2	GCAGAATTCAAGGGGTGAAGGCGCGTCTC
KO3-EcoRI	GCAGAATTCCAACCATCATCGATGAATTG-3′
KO4	CAACCCTGAAGCTTGCATGC
Tbd_0055-5	GCATCTAGATTAGATCGCCGCCTTCAACTCGC
Tbd_0055-6	CCTCGACCGACATCTCGATC
Tbd_0070-1	AAGGTCGGCATCCGCTTCAC
Tbd_0070-2	GCAGAATTCTCAGCGGGAAAAGTGCTGCATCA
Tbd_0070-5	GCATCTAGACGATCATCGACAAGCGCACG
Tbd_0070-6	GCTCTGCGTCGTATCGAAGG
Tbd_0094-1	TGCGACCGCTACGAAGTGCA
Tbd_0094-2	GCAGAATTCTGCTTCTCCTCGGAATTGAC
Tbd_0094-5	GCATCTAGACCGCATGACGAGCATGCTGA
Tbd_0094-6	GATGGCCGAGCCGAGGATGT
Tbd_0128-Tbd_0129-1	TTTCTTGAACAGGGATTGCA
Tbd_0128-Tbd_0129-2	GCAGAATTCAGGGTGCTCCAAAAAGTCGA
Tbd_0128-Tbd_0129-5	GCATCTAGAACCTTCCACGACGAGAATTG
Tbd_0128-Tbd_0129-6	TTCAACGAGGACAATTTCGG
Tbd_0137-f^b^	GGTACCAAGGATGCGTCCCTAGAGTGAAG
Tbd_0137-r^b^	GGTACCTGCAATTCCTCGACGAAATGG
Tbd_0137-Tbd_0139-1	CACGCTTCGACAATATGGAC
Tbd_0137-Tbd_0139-2	GCAGAATTCTTACACTCTAGGGACGCATCC
Tbd_0137-Tbd_0139-5	GCATCTAGAGCGGGGTATTCGAGGAAGAC
Tbd_0137-Tbd_0139-6	TAGACGAATAGCGCCGACAG
Tbd_0341-1	GTCCCTCCGACCTTCAGCAG
Tbd_0341-2	GCAGAATTCGTGCACCGAACCTGACCGAC
Tbd_0341-5	GCATCTAGATTAGCTCATTGCTTGTCCTTCGG
Tbd_0341-6	GACGATCGAGAAGGTCGACG
Tbd_0820-1	AAGCTCGCGGTCAGGTCTTG
Tbd_0820-2	GCAGAATTCAAGACCCTCGAACGGGTTGA
Tbd_0820-5	GCATCTAGATGATTTTCGCGCCAGCTGGG
Tbd_0820-6	GGGTCTTTGAGCGTCTGTTC
Tbd_0822-1	TTCTTCAACCTCGTGCTCGG
Tbd_0822-2	GCAGAATTCTTAATTGTTCTCGTGCCAGACGT
Tbd_0822-5	GCATCTAGAGCCGAACAGACGCTCAAAGA
Tbd_0822-6	AGCATGCTGCCCTGGATCA
Tbd_1357-1	TGTTCCAGGCGCCTTACTTG
Tbd_1357-2	GCAGAATTCTCCCTGTTTCTGGCTTACGC
Tbd_1357-5	GCATCTAGAGAGAGCTTCCGCAAGCCTTT
Tbd_1357-6	TCTGCTCGACCTGTGTTTGC
Tbd_1741-1	TTCATCCCCCTGTACCTCGC
Tbd_1741-2	GCAGAATTCTTACTTAGGCCTGGGCACGACGC
Tbd_1741-5	GCATCTAGATCAACCTCAACGACCTCGGT
Tbd_1741-6	TGCGCTGCTCGAGCGACTGT
Tbd_1831-1	CCAGATGTCGTTCTGGGGTG
Tbd_1831-2	GCAGAATTCTTAGCCAGTCACCCTTTCCG
Tbd_1831-5	GCATCTAGATGCGATCGTCGGTGATCTCG
Tbd_1831-6	GGCAGTTCGATGCCGTAGTG
Tbd_2026-Tbd_2027-1	CCATCGCGACGATCATGTAG
Tbd_2026-Tbd_2027-2	GCAGAATTCCTTGCTCGCTCTCTCCTCGG
Tbd_2026-Tbd_2027-5	GCATCTAGAAATACTGAGCCGAGCCCTCT
Tbd_2026-Tbd_2027-6	CGACTTCATTGAGGCGAGCT
Tbd_2060-1	CTATCACGTGCAGACCGGTG
Tbd_2060-2	GCAGAATTCTCA GCACAGCACGACGTTGAG
Tbd_2060-5	GCATCTAGAGGTGGTTCGGCGACTACAAC
Tbd_2060-6	CGATGCGCACGAGATCGAAC
Tbd_2181-1	TTGCGCAGGAACATCGCGAG
Tbd_2181-2	GCAGAATTCGAGTTCTCCAAGCATCAAAG
Tbd_2181-5	GCATCTAGACCCACCCCTGATCGAGGAGA
Tbd_2181-6	TCTCGAAGGGGACTCGATCC
Tbd_2545-1	TACTCCTTGTCCAGCAGGTG
Tbd_2545-2	GCAGAATTCGATCTTCCCATCCATCCGAT
Tbd_2545-5	GCATCTAGATCTGCAACATCAGCATGCTC
Tbd_2545-6	TCGCGATCGCCTACATCGAC
Tbd_2628-1	AGCAGCGTCGGGCCTTTCTG
Tbd_2628-2^c^	CAATTCATCGATGATGGTTGCTACATCGTGTTTTCCCCTTTGC
KO3^c^	CAACCATCATCGATGAATTG
Tbd_2628-5^c^	GCATGCAAGCTTCAGGGTTGTAGGCGAGCGGAGATGCGAG
Tbd_2628-6	TCGGTCAGCCGCGCTTTGCG

a Relevant restriction sites are underlined.

b For Tbd_0137, an insertion mutant was made according to the method described previously (Letain etal., 2007).

c Restriction sites were not included in primers for the Tbd_2628 mutant because the recombinant PCR product used to replace Tbd_2628 was generated by annealing the homologous ends of the individual PCR products.

The three digested PCR products were ligated using a Fast-Link DNA Ligation Kit (Epicentre Biotechnologies). The ligation product was used as a template for recombinant PCR, which was carried out with Primers Tbd_0055-1 and Tbd_0055-6 using the following conditions: 94°C for 30 s, followed by 35 cycles of 94°C for 30 s, 55°C for 30 s, and 72°C for 3 min and a final extension at 72°C for 10 min. The recombinant PCR product was purified and used for transformation of *T. denitrificans*, which was performed with ~100 ng of DNA according to methods described previously (Letain et al., 2007). After electroporation, cells were spread on agar plates containing 50 μg/mL kanamycin and cultured under denitrifying conditions at 30°C in an anaerobic glove box. Mutant colonies were cultured with growth medium containing 50 μg/mL kanamycin. The gene replacement was confirmed by PCR with primeTbd_0055-1 and Tbd_0055-6 using the genomic DNA from the mutant.

### CONSTRUCTION OF MUTANT STRAINS BY RANDOM TRANSPOSON MUTAGENESIS

Competent cells of *T. denitrificans* were prepared as described elsewhere (Letain et al., 2007). One μL EZ-Tn5 < KAN-2 > Tnp transposome (Epicentre Biotechnologies) was mixed with 50 μL chilled competent cells. Electroporation was performed according to previously described methods (Letain et al., 2007), with the modification that 15 kV/cm was chosen as the electroporation voltage rather than 12.5 kV/cm. After electroporation, 1 mL pre-warmed growth medium was added to the cuvette to resuspend cells and the cell suspension was transferred into a 1.5-mL microcentrifuge tube. After a 1-h recovery, kanamycin was added, and the cells were plated on solid medium containing 50 μg/mL kanamycin and incubated at 30°C in an anaerobic glove box. Negative controls included blank competent cells, blank medium, or a blank plate.

### SCREENING FOR TRANSPOSON MUTANTS DEFECTIVE IN Fe(II) OXIDATION

Colonies from plates containing kanamycin were picked into 96-well plates with 240 μL thiosulfate-limited medium in each well (the concentration of thiosulfate was 10 mM rather than the 20 mM used in growth medium). After a 72-h incubation in sealed containers in the anaerobic glove box, the OD_600_ values of the 96-well plates were measured. 100 μL of cell culture was transferred to another 96-well plate containing 100 μL 3 mM Fe(II). The initial Fe(II) concentration was determined spectrophotometrically using the Ferrozine assay described above. The 96-well plates were sealed and incubated at 30°C in the anaerobic glove box for 8 days, after which the Fe(II) concentration of the plates was again measured using the Ferrozine assay.

The mutants that displayed defective Fe(II) oxidation were cultured in regular (thiosulfate-nitrate) growth medium containing 50 μg/mL kanamycin and whole-cell suspension assays were performed as described above. Genomic DNA of the confirmed defective mutants as well as pUC19 plasmid DNA was extracted as described above and digested with SalI and XbaI (New England Biolabs). The digested genomic DNA and pUC19 plasmid DNA were purified with a QIAquick PCR Purification Kit (Qiagen) and ligated using a Fast-Link DNA Ligation Kit (Epicentre Biotechnologies) following the manufacturer’s protocols. 1 μL of the ligation product was used for electroporation of One Shot TOP10 Electrocomp *E. coli* cells (**Table 2**) followed by recovery in SOC medium (Invitrogen, Grand Island, NY, USA) according to the manufacturer’s instructions, and spread on lysogeny broth (LB) agar plates containing 50 μg/mL kanamycin. The kanamycin-resistant colonies were cultured in liquid LB medium, followed by plasmid extraction using a QIAprep Spin Miniprep Kit (Qiagen). The location of the insertion was determined by sequencing the plasmid with the KAN-2 FP-1 Forward Primer and KAN-2 RP-1 Reverse Primer, which were provided with the EZ-Tn5 < KAN-2 > Tnp Transposome Kit. DNA sequencing was performed by the DNA Sequencing Facility at the University of California, Berkeley (Berkeley, CA, USA).

## RESULTS

In this study’s search for *T. denitrificans* proteins catalyzing nitrate-dependent Fe(II) oxidation, both targeted and random mutagenesis strategies were undertaken. The choice of gene candidates for targeted mutagenesis was based in part on the results of whole-genome transcriptional studies. Briefly, criteria for targeting genes included the following: (i) *c*-type cytochromes shown to be involved in nitrate-dependent U(IV) oxidation and other membrane-associated *c*-type cytochromes of unknown function, (ii) *c*-type cytochromes upregulated during nitrate-dependent Fe(II) oxidation and/or highly expressed under relevant conditions, (iii) any genes (not just *c*-type cytochromes) strongly upregulated under nitrate-dependent Fe(II)-oxidizing conditions.

Accordingly, after presenting data on the physiology of nitrate-dependent Fe(II) oxidation in *T. denitrificans*, the Section “Results” covers the following topics: (a) gene expression trends among *c*-type cytochromes, (b) genes highly upregulated under Fe(II)- and U(IV)-oxidizing conditions, (c) determination of Fe(II)-oxidizing activity in strains with targeted mutations, and (d) random transposon mutants defective in Fe(II) oxidation.

### ANAEROBIC, NITRATE-DEPENDENT Fe(II) OXIDATION IN *T. denitrificans*

**FIGURE 1:**
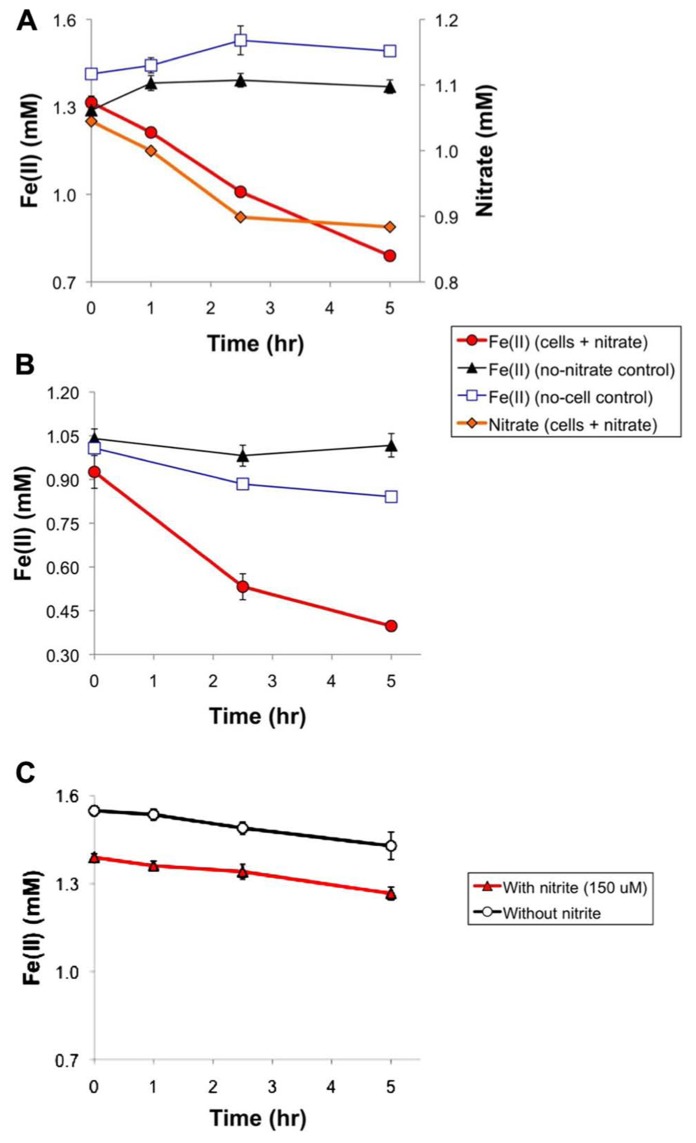
**Anaerobic cell suspension studies of nitrate-dependent Fe(II) oxidation by wild-type *T. denitrificans* and related abiotic studies. (A)** Cell suspensions with the headspace consisting of the standard glove box atmosphere (nominally 85% N_2_ – 10% CO_2_ – 5% H_2_), **(B)** cell suspensions with ultra high-purity N_2_ in the headspace, and **(C)** abiotic studies (without *T. denitrificans* cells) under the same conditions as in **(A)** but with 150 μM nitrite (the typical amount of nitrate consumed in cell suspension experiments) replacing 1 mM nitrate. The OD_600_ of the experiments represented by **(A)** and **(B)** was 1.8. Data points represent the averages of triplicates and error bars represent one standard deviation.

Although Straub et al. (1996) reported that *T. denitrificans* (ATCC 25259, the same strain used in this study) catalyzed autotrophic, nitrate-dependent Fe(II) oxidation, they did not show any data in support of this finding. In this section, we present some data to reveal important characteristics of this process. **Figure 1A** displays two aspects of nitrate dependence in Fe(II) oxidation by *T. denitrificans*: (1) *T. denitrificans* cells will oxidize Fe(II) in the presence of nitrate but not in its absence and (2) at least initially, there is a strong correlation between Fe(II) oxidation and nitrate reduction. Regarding the latter point, over the first 2.5 h, a linear regression of Fe(II) oxidation vs. nitrate consumption had an *r*^2^ value of 0.999 and a slope of 2.08 for the experiment represented in **Figure 1A**. The slope of approximately 2 suggests that nitrate was reduced only to nitrite during this period. Subsequently, this ratio increased as nitrate consumption decreased while Fe(II) oxidation continued, suggesting that nitrite was probably serving as an electron acceptor for further Fe(II) oxidation. Nitrite was not detected in such Fe(II) oxidation studies (at a detection limit of 150 μM, accounting for dilution), which may not be surprising because the total amount of nitrate reduced in such experiments was in the detection limit range (160 μM for the experiment represented in **Figure 1A**).

In light of extensive discussions in the literature about the potential role that nitrite and other denitrification intermediates could play in abiotic oxidation during nitrate-dependent Fe(II) oxidation (Carlson et al., 2012; Picardal, 2012; Kopf et al., 2013), we carried out abiotic studies of anaerobic Fe(II) oxidation in the presence of 150 μM nitrite (approximately the maximum amount of nitrite that could be produced in our studies, based on total nitrate consumption) and in the absence of nitrate. Such studies (**Figure 1C**) revealed negligible Fe(II) oxidation caused by nitrite under our assay conditions. 120 μM of Fe(II) was oxidized in both the presence *and* absence of 150 μM nitrite (**Figure 1C**). Thus, abiotic oxidation by nitrite probably accounts for a small portion of the ~500 μM Fe(II) typically oxidized in our experiments (e.g., for the experiments represented in Figures 1A, B).

One noteworthy difference between nitrate-dependent Fe(II) and U(IV) oxidation by *T. denitrificans* is that U(IV) oxidation appears to require H_2_ to proceed (Beller, 2005), whereas Fe(II) oxidation does not (**Figure 1B**). In the experiment represented in **Figure 1B**, only N_2_ was present in the headspace, whereas in the experiment represented in **Figure 1A**, the headspace consisted of a mixture of N_2_/CO_2_/H_2_ (see Materials and Methods). The same amount of Fe(II) was oxidized either with or without H_2_ in the headspace (~500 μM).

These studies show that Fe(II) can be the sole electron donor for *T. denitrificans* during nitrate-dependent Fe(II) oxidation, in contrast to the organotrophic Fe(II) oxidizers that require an electron donor, such as acetate, in addition to Fe(II). However, we did not find evidence that nitrate-dependent Fe(II) oxidation could support growth of *T. denitrificans* in liquid medium over a 20-day period.

### GENE EXPRESSION TRENDS AMONG *c*-TYPE CYTOCHROMES

For reasons discussed previously [e.g., the relatively high reduction potential of some *c*-type cytochromes; a number of *c*-type cytochromes identified as having a role in anaerobic U(IV) and Fe(II) oxidation], *c*-type cytochromes are primary candidates for catalyzing nitrate-dependent Fe(II) oxidation in *T. denitrificans*. The genome of *T. denitrificans* encodes more than 50 predicted proteins with the CXXCH heme-binding motif, many of which are mono and diheme *c*-type cytochromes (Beller et al., 2006a). However, BLASTP searches (Altschul et al., 1997) reveal that the genome does not encode homologs of *c*-type cytochromes that have been associated with Fe(II) oxidation in other bacteria, such as *pioA* (Jiao and Newman, 2007) or *foxE* (Croal et al., 2007; Saraiva et al., 2012) in anoxygenic phototrophic bacteria, *mtoA* in *Sideroxydans lithotrophicus* ES-1 (Liu et al., 2012), or *cyc2* in *Acidithiobacillus* *ferrooxidans* (Castelle et al., 2008). The genome also does not encode proteins associated with these *c*-type cytochromes, such as PioB, PioC, FoxY, FoxZ, MtoB, or CymA_ ES-1_.

**FIGURE 2:**
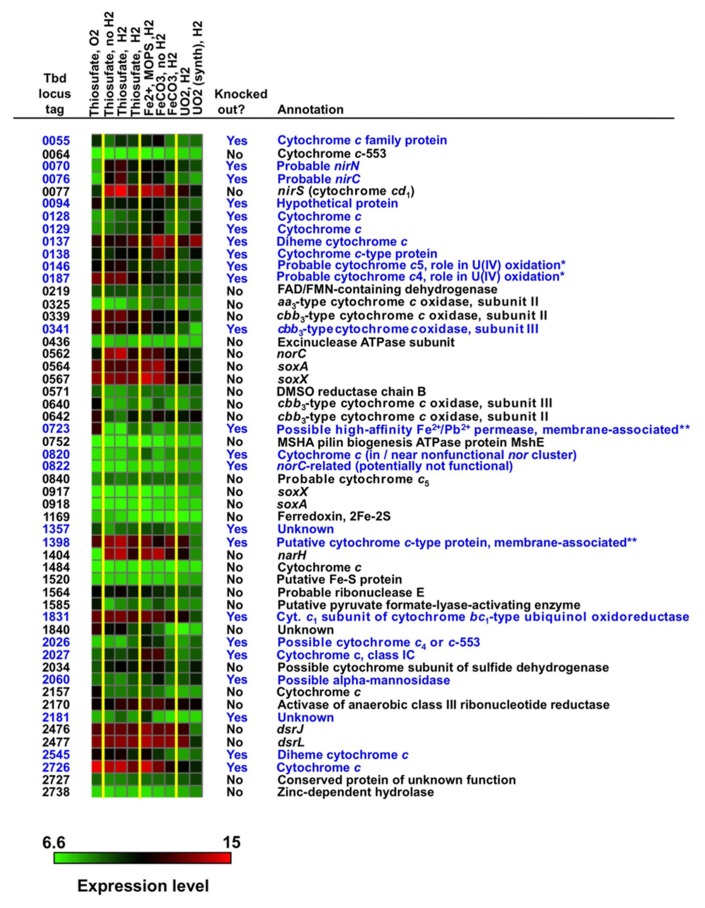
**Expression profiles and annotations of genes encoding proteins with the CXXCH heme-binding motif, many of which are *c*-type cytochromes (see text).** The log_2_ intensity data plotted in this figure were normalized as described and represent, with one exception, the average of three biological replicates and three technical replicates (see text). The exposure conditions for the arrays (displayed directly above the heat map) listed from left to right are the same as the conditions listed in **Table 1** from top to bottom. Genes highlighted in blue were targeted for mutation studies. An intensity color scale is shown. To provide some perspective for the intensity scale, the following are the average percentile values for intensity for all genes on all arrays: 50th percentile = 8.0, 75th percentile = 9.0, 90th percentile = 10.4, 95th percentile = 11.3, and 100th percentile = 15.1. The symbol “*” represents genes previously shown to be associated with nitrate-dependent U(IV) oxidation (Beller et al., 2009) and “**” represents genes encoding proteins that were experimentally shown to be membrane associated (see text).

Expression data for genes encoding predicted proteins with the CXXCH motif (including *c*-type cytochromes) are presented in **Figure 2**. The log_2_ intensity data plotted in **Figure 2** were normalized as described in Section “Materials and Methods” and represent, with one exception, the average of three biological replicates, each of which had three technical on-chip replicates. The exception is the “UO_2_ (synth.)” column, which represents only one biological replicate. The exposure conditions for the arrays (displayed directly above the heat map) listed from left to right are the same as the conditions listed in **Table 1** from top to bottom.

Some of the most highly expressed *c*-type cytochromes under a range of conditions [including thiosulfate and Fe(II) oxidation under denitrifying conditions] have known functions associated with denitrification or S-compound oxidation, including *nirS* (Tbd_0077), *norC* (Tbd_0562), *soxA* (Tbd_0564), *soxX* (Tbd_0567), and *dsrJ* (Tbd_2476). Further, Tbd_1831 is a cytochrome *c*_1_ subunit of complex III (cytochrome *bc*_1_-type ubiquinol oxidoreductase). However, other highly expressed, putative *c*-type cytochromes have unknown functions, including Tbd_0137 (which is clustered in the genome with other *c*- and *b*-type cytochromes), Tbd_1398, and Tbd_2726. Note that *narH* (Tbd_1404; ß subunit of the respiratory nitrate reductase) was highly expressed but is not a *c*-type cytochrome – it is an Fe-S protein. Open reading frames (ORFs) shown in blue in **Figure 2** were chosen as targets for insertion or replacement mutations. Among the more highly expressed genes that were *not* chosen as targets were *nirS*, *norC*, and *narH* (because these were thought to be essential genes under denitrifying conditions) and *soxA*, *soxX*, and *dsrJ* (whose S-compound oxidation functions are well known). More detailed discussion of target selection is presented later.

Additional information footnoted in **Figure 2** merits mention here. Two diheme *c*-type cytochromes included in the figure, Tbd_0146 and Tbd_0187, were experimentally shown to play a role in nitrate-dependent U(IV) oxidation in *T. denitrificans* using insertion mutations and complementation in *trans* (Beller et al., 2009). These two proteins, along with Tbd_0723 and Tbd_1398, were experimentally shown to be membrane associated (Beller et al., 2009). Sucrose-density-gradient ultracentrifugation experiments were intended to identify OM proteins [which might be expected to directly contact insoluble U(IV) or Fe(II) phases], but the density separation was clearly not specific to only OM proteins and one can only claim that Tbd_0146, Tbd_0187, Tbd_0723, and Tbd_1398 are membrane-associated, not necessarily OM proteins (Beller et al., 2009). As membrane-associated *c*-type cytochromes of unknown function, all four of these proteins were clearly of interest as candidates for catalyzing anaerobic Fe(II) oxidation.

**FIGURE 3:**
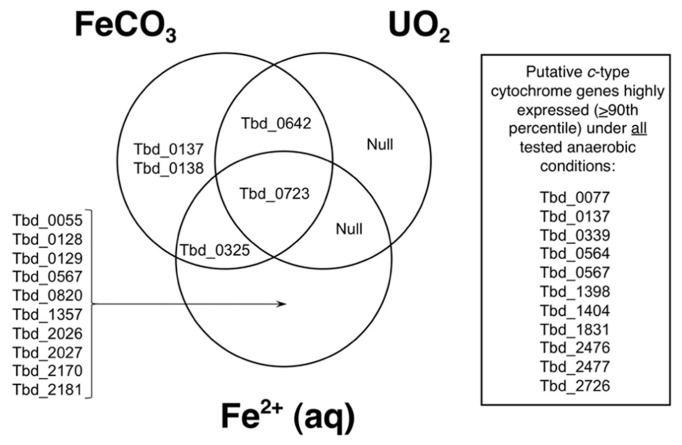
**Venn diagram showing genes from **Figure 2** (mostly *c*-type cytochromes) that are at least twofold upregulated relative to control conditions (thiosulfate and H_**2**_; Group VII) at *P *≤ 0.0001.** All exposure conditions shown include H_2_, and consist of Group V (FeCO_3_ and H_2_), Group VI (UO_2_ and H_2_), and Group II [Fe^2^^+^ (aq) and H_2_]. Also shown are genes from Figure2 that are highly expressed under all anaerobic conditions tested (see text).

Upregulation of genes encoding predicted proteins with the CXXCH motif is represented in **Figure 3**. In this Venn diagram, genes are listed that are at least twofold upregulated relative to control conditions (thiosulfate and H_2_; Group VII) at *P *≤ 0.0001. All exposure conditions shown include H_2_, and consist of Group V (FeCO_3_ and H_2_), Group VI (UO_2_ and H_2_), and Group II [Fe^2^^+^ (aq) and H_2_]; recall that, in *T. denitrificans*, H_2_ is required for nitrate-dependent U(IV) oxidation (Beller, 2005). Looking across the entire genome, 476 genes were upregulated at least twofold under these conditions, and for putative *c*-type cytochromes, 15 genes met this twofold criterion. The locus tags for these upregulated genes are identified in **Figure 3**. The largest number of upregulated genes was for nitrate-dependent, Fe^2^^+^-oxidizing conditions (12 genes total, with 10 genes unique to this exposure condition). The single gene that was upregulated at least twofold under all three conditions shown was Tbd_0723, which is putatively a Fe^2^^+^ permease that is located in a large gene cluster associated with iron acquisition (Beller et al., 2006b). No putative *c*-type cytochrome genes were uniquely upregulated under nitrate-dependent, UO_2_-oxidizing conditions (**Figure 3**). Most of the upregulated putative *c*-type cytochrome genes were targeted for insertion mutations (see blue highlighted ORFs in **Figure 2**). Those that were *not* targeted included Tbd_0567 (*soxX*, discussed earlier), Tbd_2170 (which is not a cytochrome, but rather an Fe-S protein that is an activase for anaerobic ribonucleoside triphosphate reductase, a glycyl radical enzyme), Tbd_0325 (subunit II of *aa*_3_-type cytochrome *c* oxidase), and Tbd_0642 (subunit II of *cbb*_3_-type cytochrome *c* oxidase, CcoO). Also shown in **Figure 3** are putative *c*-type cytochrome genes that were highly expressed (>90th percentile expression) under all anaerobic conditions tested (including the control, thiosulfate-oxidizing conditions). Most of these genes have already been addressed except Tbd_0339, which is a homolog of Tbd_0642 (these two share 51% amino acid sequence identity).

## A COMMON GROUP OF UPREGULATED GENES UNDER Fe(II)- AND U(IV)-OXIDIZING CONDITIONS

**FIGURE 4:**
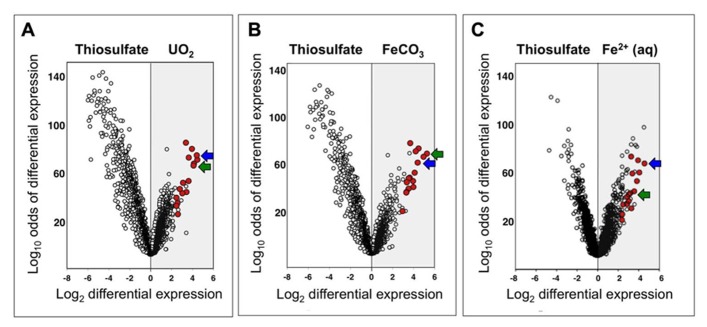
**Volcano plots of log_10_ odds of differential expression versus log_2_ fold differential expression for all *T. denitrificans* genes under nitrate-dependent UO_2_-oxidizing (A), FeCO_3_-oxidizing (B), and Fe^2^^+^-oxidizing (C) conditions (Groups VI, V, and II, respectively; **Table 1**) relative to nitrate-dependent thiosulfate-oxidizing conditions (Group VII)**. A highly upregulated group of 16 genes is highlighted in red (see text). Two of the most highly upregulated genes are indicated with blue arrows (Tbd_2628) and green arrows (Tbd_1948).

The search for enzyme candidates for nitrate-dependent Fe(II) oxidation extended beyond *c*-type cytochromes to any genes that were highly upregulated under conditions of interest. Data analysis revealed that a common group of genes was upregulated under nitrate-dependent FeCO_3_-, Fe^2^^+^-, and UO_2_-oxidizing conditions (Groups V, II, and VI, respectively; **Table 1**) relative to nitrate-dependent thiosulfate-oxidizing conditions (Group VII). Of the top 25 upregulated genes under each of the three conditions of interest (i.e., FeCO_3_-, Fe^2^^+^-, and UO_2_-oxidizing), a group of 16 genes belonged to all three top-25 groups. These 16 genes are shown in red in **Figure 4**, a volcano plot, which graphs log_10_ odds of differential expression vs. log_2_ fold differential expression for all 2,832 ORFs identified in the draft genome at the time of microarray design (the finished genome is annotated to have 2,827 ORFs; Beller et al., 2006a). Upregulation under FeCO_3_-, Fe^2^^+^-, and UO_2_-oxidizing conditions is plotted in the gray regions of Figures 4A– C, and the ORFs most highly upregulated relative to thiosulfate-oxidizing conditions are plotted furthest to the right. The group of 16 genes clearly contains many of the most highly upregulated genes under all three conditions, ranging from 5- to 39-fold upregulation and averaging 13-fold upregulation relative to thiosulfate-oxidizing conditions.

**FIGURE 5:**
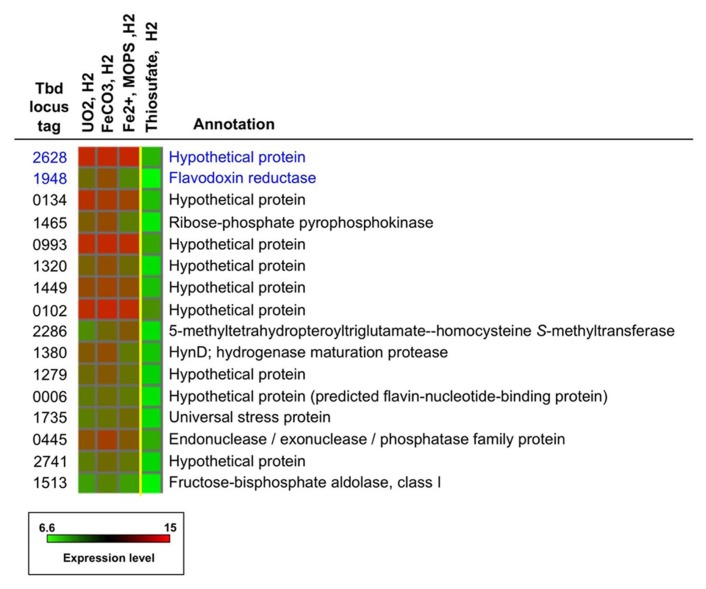
**Expression profiles and annotations for a group of 16 of the most highly expressed genes under under nitrate-dependent UO_2_-, FeCO_3_-, and Fe^2^^+^-oxidizing conditions (Groups VI, V, and II, respectively; see text)**. The two genes highlighted in blue were targeted for mutation studies. The intensity color scale is the same as for **Figure2**.

The identities (locus tag numbers and annotation) of the 16 highly upregulated genes under Fe(II)- and U(IV)-oxidizing conditions are shown in **Figure 5**, along with the log_2_ intensity data for expression of these genes under relevant conditions. The 16 genes are listed in decreasing order of geometric-average, fold upregulation for nitrate-dependent FeCO_3_-, Fe^2^^+^-, and UO_2_-oxidizing conditions relative to nitrate-dependent thiosulfate-oxidizing conditions [ranging from 22-fold upregulation (Tbd_2628) to 6.6-fold upregulation (Tbd_1513)]. In some cases, absolute expression levels are very high in addition to relative expression levels. This is particularly true for Tbd_2628, which had log_2_ intensities of 13.1 to 13.3 under the non-control conditions shown (well above the 95th percentile value of 11.3 for all genes on all arrays). Note that the top-ranked two genes are indicated in **Figure 4** with blue arrows (Tbd_2628) and green arrows (Tbd_1948). Both of these genes were targeted for insertion/replacement mutations.

As shown in **Figure 5**, many (nine) of the 16 genes shown encode proteins of unknown function (hypothetical proteins), including Tbd_2628. Tbd_1948 is annotated as a flavodoxin reductase and thus may have a function related to electron transfer. Other encoded proteins on the list may also have some role in electron transfer, including Tbd_0006 (a predicted flavin-nucleotide-binding protein) and Tbd_0993 (located upstream of a ubiquinone biosynthesis gene, Tbd_0994). As might be expected, some of these genes may be associated with stress response (Tbd_1735) and metal efflux (e.g., Tbd_1320 is located near a gene cluster associated with copper resistance, Tbd_1324-1326; Beller et al., 2006a; and Tbd_2741 is located next to a gene annotated as a heavy metal-translocating P-type ATPase, Tbd_2740). A noteworthy gene listed in **Figure 5** is Tbd_1380, *hynD*, a hydrogenase maturation protease that is the first gene transcribed in an operon that encodes a periplasmic group 1 [NiFe]hydrogenase (Beller et al., 2006a). This is of particular interest because, as noted previously, *T. denitrificans* only carries out nitrate-dependent U(IV) oxidation when H_2_ oxidation is occurring (Beller, 2005). However, the gene encoding the large (active-site-bearing) subunit of the associated hydrogenase (Tbd_1375, *hynL*) was previously knocked out (Letain et al., 2007) and the resulting mutant strain was not defective in nitrate-dependent U(IV) oxidation (H. R. Beller, unpub-lished data).

### EFFECTS OF TARGETED MUTATIONS ON Fe(II) OXIDATION

In total, 26 insertion or replacement mutants were tested in assays for anaerobic, nitrate-dependent Fe(II) oxidation (**Table 4**). The specific Fe(II) oxidation activities of these mutant strains are listed in **Table 4** along with the activity of the wild-type (for comparison) and the rationale for targeting the genes listed. The specific activities are based on averages of at least three biological replicates; all experiments included both no-nitrate and no-cell controls. In some cases, multiple experiments were performed to confirm results. Of strains with targeted mutations, none was more than 15% defective relative to the wild-type. Of particular note is the observation that the two *c*-type cytochrome mutants that were found to be ~50% defective in nitrate-dependent U(IV) oxidation, ΔTbd_0146 and ΔTbd_0187 (Beller et al., 2009), were not at all defective in nitrate-dependent Fe(II) oxidation, nor was the double-mutant strain (**Table 4**).

**Table 4:** Specific rates of nitrate-dependent Fe(II) oxidation by *T. denitrificans* wild-type and mutants under study.

Mutant strain^a^	Fe(II) oxidation rate	Rationale for targeting ORF Mean ± SD (μmol/min/mg protein)
Tbd_0055	3.1 ± 0.10	Upregulated *c*-type cytochrome
Tbd_0070	3.2 ± 0.21	Moderately expressed *c*-type cytochrome
Tbd_0076	^b^	Moderately expressed *c*-type cytochrome
Tbd_0094	2.9 ± 0.10	Protein with CXXCH motif and unknown function
Tbd_0128-Tbd_0129	4.8 ± 0.63	Upregulated *c*-type cytochromes (both diheme)
Tbd_0137	4.2 ± 0.03	Highly expressed and upregulated *c*-type cytochrome (diheme)
Tbd_0137-Tbd_0139	3.1 ± 0.13	Highly expressed *c*- and *b*-type cytochromes
Tbd_0146	3.7 ± 0.09	Diheme *c*-type cytochrome with proven role in U(IV) oxidation (Beller etal., 2009)
Tbd_0187	4.7 ± 0.20	Diheme *c*-type cytochrome with proven role in U(IV) oxidation (Beller etal., 2009)
Tbd_0146 Tbd_0187	4.8 ± 0.63	Double mutant of Tbd_0146 and Tbd_0187
Tbd_0341	2.8 ± 0.19	Moderate expression *cbb*_3_-type cytochrome *c* oxidase, subunit III
Tbd_0723	3.3 ± 0.30	Upregulated with UO_2_, FeCO_3_, and Fe^2^^+^
Tbd_0820	4.0 ± 0.28	Upregulated *c*-type cytochrome (diheme)
Tbd_0822	5.2 ± 0.12	Non-canonical *norC* (diheme; Beller etal., 2006a)
Tbd_1145	2.1 ± 0.08	Random transposon mutant found to be defective in nitrate-dependent Fe(II) oxidation
Tbd_1357	3.2 ± 0.17	Protein with CXXCH motif and unknown function
Tbd_1398	3.8 ± 0.07	Highly expressed *c*-type cytochrome
Tbd_1741	4.8 ± 0.16	Highly expressed homolog of protein in Fe(II)-oxidizing *Mariprofundus ferrooxydans* PV-1^c^
Tbd_1831	4.1 ± 0.19	Highly expressed cytochrome *c*_1_ subunit of cytochrome *bc*_1_-type ubiquinol oxidoreductase
Tbd_1948	4.6 ± 0.20	Highly upregulated with UO_2_, FeCO_3_, and Fe^2^^+^
Tbd_2026-Tbd_2027	3.3 ± 0.13	Upregulated *c*-type cytochromes
Tbd_2060	3.0 ± 0.32	Upregulated protein with CXXCH motif
Tbd_2181	3.3 ± 0.22	Protein with CXXCH motif and unknown function
Tbd_2545	4.7 ± 0.43	Moderately expressed *c*-type cytochrome (diheme)
Tbd_2628	2.9 ± 0.36	Highly upregulated with UO_2_, FeCO_3_, and Fe^2^^+^
Tbd_2726	3.3 ± 0.35	Highly expressed *c*-type cytochrome
**Wild-type**	3.3 ± 0.52	
ATCC25259		

a Insertion or replacement mutation was made for gene(s) with indicated locus tag(s).

b Slow growth precluded performing an assay with this strain (this gene, located in the *nir* operon, may be essential for denitrification).

c Tbd_1741 has 31% amino acid sequence identity to SPV1_03948, a putative molybdopterin protein in *Mariprofundus ferrooxydans* PV-1 that is expressed during Fe(II) oxidation by that strain (Singer etal., 2011). In *T. denitrificans*, this gene is part of a cluster (Tbd_1739-1742) that is highly expressed under denitrifying conditions (data not shown).

### RANDOM TRANSPOSON MUTANTS DEFECTIVE IN Fe(II) OXIDATION

More than 20,000 random transposon mutants were screened for nitrate-dependent Fe(II) oxidation. The most reproducibly defective strain had a mutation in *nuoD* (Tbd_1145), which encodes the D subunit of NADH:ubiquinone oxidoreductase (complex I). Fe(II) oxidation assays revealed that this mutant was ~35% defective in Fe(II) oxidation relative to the wild-type (**Table 4**). Notably, it was ~33% defective in nitrate reduction in an independent positive control assay in which thiosulfate was the electron donor rather than Fe(II). These results are consistent with both (a) the expected role of complex I in transferring electrons to the ubiquinone pool, and ultimately, to nitrate reductase via ubiquinol and (b) the tight coupling between Fe(II) oxidation and nitrate reduction. In effect, the mutation in *nuoD* is likely causing a direct defect in delivery of reducing equivalents to nitrate reductase and is thus indirectly causing a defect in nitrate-dependent Fe(II) oxidation.

## DISCUSSION

Although we were able to demonstrate that there is a strong linkage between Fe(II) as the sole electron donor and nitrate reduction to nitrite, and that abiotic interactions of nitrite with Fe(II) contribute little, if anything, to this process, we have no evidence that this linkage results in energy conservation or growth. It is clear that nitrate-dependent Fe(II) oxidation in *T. denitrificans*, unlike nitrate-dependent U(IV) oxidation, has no co-metabolic dependence on H_2_ oxidation and is not catalyzed by the diheme *c*-type cytochromes Tbd_0146 and Tbd_0187. In fact, none of the *c*-type cytochrome genes targeted in this study was shown to be important to nitrate-dependent Fe(II) oxidation. Possible explanations include the following: (a) one or more *c*-type cytochromes are involved in nitrate-dependent Fe(II) oxidation in *T. denitrificans*, but we did not target the appropriate genes in this study, (b) since the *T. denitrificans* genome encodes many *c*-type cytochromes, one or more *c*-type cytochromes are being upregulated in mutant strains to compensate for the loss of the targeted *c*-type cytochrome, or (c) *c*-type cytochromes are not involved in nitrate-dependent Fe(II) oxidation in *T. denitrificans*.

The only significantly defective mutant identified in this study had a mutation in *nuoD* (Tbd_1145) of NADH-ubiquinone oxidoreductase (complex I in the electron transport chain); this strain was equally defective in nitrate-dependent Fe(II) oxidation and nitrate-dependent thiosulfate oxidation (tested independently). In a sense, this can be easily rationalized in that complex I mediates transfer of electrons derived from NADH + H^+^ oxidation to the quinone pool (ubiquinone), which can then pass these electrons to the *b*-cytochrome (NarI) subunit of nitrate reductase (NarGHI; Berks et al., 1995; Zumft, 1997; Richardson, 2000). A mutation in complex I would presumably preclude this electron transfer to nitrate reductase and create the observed defective phenotypes. However, the source of NADH is problematic in light of the electron donor involved, namely, Fe(II). The reduction potential of the Fe(III)-NTA/Fe(II)-NTA couple, which is probably most relevant to our Fe(II) oxidation assay, is very high: +0.372 V (pH 7; Ilbert and Bonnefoy, 2013). This reduction potential is still exergonic in combination with nitrate reduction to nitrite (the ${NO}_{3}^{-}/{NO}_{3}^{-}$reduction potential is 0.42 V at pH 7; Ilbert and Bonnefoy, 2013) but is far too high to result in direct NADH production during nitrate-dependent Fe(II) oxidation. Thus, it seems that reverse electron transfer would be required to produce NADH to feed into complex I and ultimately reduce nitrate. A reverse electron transfer pathway proposed for the aerobic, acidophilic Fe(II)-oxidizing bacterium, *Acidithiobacillus ferrooxidans*, involves transfer of some portion of Fe(II)-derived electrons “uphill” (i.e., in a thermodynamically unfavorable direction) through complex III, ubiquinone, and complex I to produce NADH (Bird et al., 2011 and references therein). If this reverse electron transfer mechanism *via* complex III (cytochrome *bc*_1_ complex) is accurate and relevant to *T. denitrificans*, it is difficult to explain our finding that a mutant in the *c*_1_-cytochrome subunit of the cytochrome *bc*_1_ complex (Tbd_1831) was not at all defective in Fe(II) oxidation (note that the Tbd_1831 gene replacement was confirmed by PCR analysis of cells from the *in vivo* assay using Primers Tbd_1831-1 and Tbd_1831-6 and sequencing of the amplicon). Thus, the question arises: how is the NADH that is oxidized in complex I actually produced during Fe(II) oxidation? It is possible, but speculative, that reverse electron transfer in *T. denitrificans* is routed through a protein complex other than complex III (see below). It is also possible that another *c*-type cytochrome is upregulated in the Tbd_1831 mutant and is compensating for Tbd_1831’s role in complex III. However, a BLASTP search of the *T. denitrificans* genome reveals that there is no protein encoded by the genome that matches Tbd_1831 even at a low-stringency E-value of 10^-^^2^. Another possibility is that reverse electron transfer indeed functions *via* complex III in *T. denitrificans* but that the *c*_1_-cytochrome subunit is not essential for this process. Finally, it is not plausible that H_2_ oxidation could be contributing to reduction of the quinone pool, as nitrate-dependent Fe(II) oxidation takes place in the absence of H_2_ (**Figure 1B**) and the rate of Fe(II) oxidation in the *nuoD* (Tbd_1145) mutant in the absence of H_2_ is comparable to that in the presence of H_2_ (data not shown).

Regarding an alternate pathway for reverse electron transfer that does not involve complex III, it is possible that nitric oxide reductase (NorBC; Tbd_0561-Tbd_0562) could play the role that has been assigned to the cytochrome *bc*_1_ complex. Like the cytochrome *bc*_1_ complex, NorBC is a cytoplasmic-membrane complex that includes *b*- and *c*-type cytochromes [with relatively high midpoint reduction potentials in NorBC of +345 and +310 mV for low-spin *b*- and *c* hemes, respectively (Watmough et al., 2009)]. Although, unlike the cytochrome *bc*_1_ complex and qNOR nitric oxide reductases (de Vries et al., 2003; Tavares et al., 2006), NorBC is not thought to interact directly with the quinone pool, electron transfer interactions between nitric oxide reductase and the ubiquinone pool through a mediator have been suggested for other bacteria (Bell et al., 1992). Considering that mechanistic knowledge of reverse electron transfer in chemolithotrophic bacteria is currently quite limited, exploration of alternatives to complex III in a genetically tractable organism, such as *T. denitrificans*, could substantially improve understanding of this fundamental physiological process.

The above discussion and finding that a mutant in the *c*_1_-cytochrome subunit of the cytochrome *bc*_1_ complex was not defective in Fe(II) oxidation is relevant to a recently postulated mechanism for nitrate-dependent Fe(II) oxidation (Carlson et al., 2012), namely, electron transfer from Fe(II) to the quinone pool mediated by the cytochrome *bc*_1_ complex. Our results for the Tbd_1831 mutant are not consistent with this hypothesized mechanism. Further study will be required to address the role of complex III in reverse electron transfer in *T. denitrificans* and to elucidate the enzyme(s) that initiate the process of nitrate-dependent Fe(II) oxidation in this and other bacterial species.

## AUTHOR CONTRIBUTIONS

Harry R. Beller was the primary author, conceived of the overall study, and designed and participated in the microarray experiments. Peng Zhou created all replacement mutants, conducted the entire transposon mutagenesis study, and performed all Fe(II) oxidation assays. Harry R. Beller and Peng Zhou contributed equally to the manuscript. Tina C. Legler created all insertion mutants and participated in microarray studies along with Staci Kane and Tracy E. Letain. Anu Chakicherla performed statistical analyses of microarray studies. Peggy A. O’Day provided geochemical input.

## Conflict of Interest Statement

The authors declare that the research was conducted in the absence of any commercial or financial relationships that could be construed as a potential conflict of interest.
